# Case Report: Progressive central conducting lymphatic abnormalities in the RASopathies. Two case reports, including successful treatment by MEK inhibition

**DOI:** 10.3389/fgene.2022.1001105

**Published:** 2022-09-27

**Authors:** Kristiana Gordon, Matthew Moore, Malou Van Zanten, Julian Pearce, Maxim Itkin, Brendan Madden, Lakshmi Ratnam, Peter S. Mortimer, Rani Nagaraja, Sahar Mansour

**Affiliations:** ^1^ Lymphovascular Research Unit, Molecular and Clinical Sciences Research Institute, University of London, London, United Kingdom; ^2^ Lymphovascular Clinic, St. George’s University Hospitals NHS Foundation Trust, London, United Kingdom; ^3^ Cardiovascular Department, St. George’s University Hospitals NHS Foundation Trust, London, United Kingdom; ^4^ Division of Interventional Radiology, Hospital of the University of Pennsylvania, Philadelphia, PA, United States; ^5^ Department of Interventional Radiology, St. George’s University Hospitals NHS Foundation Trust, London, United Kingdom; ^6^ Gastroenterology Department, St. George’s University Hospitals NHS Foundation Trust, London, United Kingdom; ^7^ SW Thames Regional Genetics Service, St. George’s University Hospitals NHS Foundation Trust, London, United Kingdom

**Keywords:** chylous, lymphoedema, chylothorax, chylopericardium, noonan syndrome, lymphedema, genital oedema

## Abstract

The RASopathies are a group of genetic conditions resulting from mutations within the RAS/mitogen-activated protein kinase (RAS-MAPK) pathway. Lymphatic abnormalities are commonly associated with these conditions, however central conducting lymphatic abnormalities (CCLA) have only recently been described. CCLAs may be progressive and can result in devastating systemic sequelae, such as recurrent chylothoraces, chylopericardium and chylous ascites which can cause significant morbidity and even mortality. Improvements in imaging modalities of the central lymphatics have enhanced our understanding of these complex abnormalities. Management is challenging and have mainly consisted of diuretics and invasive mechanical drainages. We describe two adult males with Noonan syndrome with a severe and progressive CCLA. In one patient we report the therapeutic role of targeted molecular therapy with the MEK inhibitor ‘Trametinib’, which has resulted in dramatic, and sustained, clinical improvement. The successful use of MEK inhibition highlights the importance of understanding the molecular cause of lymphatic abnormalities and utilising targeted therapies to improve quality of life and potentially life expectancy.

## Introduction

The RASopathies are a group of rare genetic conditions caused by germline mutations in genes of the RAS/mitogen-activated protein kinase (RAS-MAPK) pathway. They occur in approximately 1/1000 live births, with no gender predilection (Rauen, 2013). These autosomal dominant disorders have genetic heterogeneity and overlapping clinical features. Characteristic features include low-set, posteriorly rotated ears, hypertelorism, ptosis, short stature, congenital heart defects, webbed neck, and down-slanting palpebral fissures (Roberts et al., 2013). Lymphatic abnormalities are recognised as a rare, but significant, feature of Noonan syndrome and other RASopathies (Tartaglia et al., 2010; Joyce et al., 2015).

The RASopathies may present antenatally with an increased nuchal translucency (NT) and persistent nuchal fold, hydrothoraces and/or fetal hydrops, consistent with abnormalities of lymphatic development. The presence of neck webbing in these conditions is considered a post-natal manifestation of *in utero* oedema, as are low-set ears, low hairline, epicanthic folds and ptosis (Opitz., 1986). Post-natal lymphatic problems in the RASopathies (including Noonan syndrome) include congenital or childhood onset bilateral lower limb lymphoedema, genital swelling with chylous reflux (i.e., the backflow of chyle from the intestinal lymphatics resulting from incompetent lymphatic vessels and valves) with leaking of ‘milky’ and sometimes blood-stained fluid from cutaneous lymphangiectasia (lymph-filled skin lymphatics looking like “blisters”) in the scrotum or labia, and systemic involvement including intestinal lymphangiectasia, chylopericardium, chylous ascites and chylothoraces (Witt et al., 1987; Joyce et al., 2015).

Investigation of lower limb lymphoedema in the RASopathies with standard radionuclide lymphoscintigraphy demonstrates lymphatic reflux and/or abnormal rerouting of lymphatic drainage (Joyce et al., 2015).

The prevalence of lymphatic problems in the RASopathies appears to be greater than originally suspected. One cross-sectional cohort study of 35 Noonan syndrome patients identified a 49% prevalence of limb lymphoedema (Smpokou., 2012) another suggests that the lifetime prevalence of lymphatic abnormalities was higher than 20% (Sleutjes et al., 2022). We published an observational study of 11 patients with a RASopathies (Joyce et al., 2015). The onset of bilateral lower limb lymphoedema was in childhood or adulthood (age of onset range 4–44 years), there was genital oedema (82%) and chylous reflux with lymphorrhoea i.e. leakage of milky lymphatic fluid from cutaneous lymphangiectasia (73%). There is no observed genotype-phenotype correlation. Six of the 11 patients had gain of function mutations in *PTPN11* (55%), 2 in *KRAS* (18%), 2 in *RIT1* (18%) and one in *BRAF* (9%)*.* This spectrum of mutations roughly correlates with that seen in a larger cohort of patients with RASopathies (Roberts., 2001).

Systemic lymphatic anomalies in the RASopathies include intestinal lymphangiectasia (IL), chylothoraces, chylopericardium and chylous ascites. Recent reports have described these as a central conducting lymphatic anomalies/ abnormalities (CCLA), defined/characterised by thoracic duct dysfunction, dilated lymphatic channels and lymphatic vessel dysmotility, resulting in abnormal drainage of lymphatic fluid and retrograde flow/reflux of lymph (Clemens et al., 2015; Trenor and Chaudry., 2014; Li et al., 2018).

Traditional radionuclide lymphoscintigraphy is inadequate for imaging the central lymphatic system. Fluoroscopic lipiodol lymphangiography and dynamic contrast enhanced MR-lymphangiography (DCMRL) are both increasingly being employed to visualise the thoracic duct and central lymphatic system. Biko et al undertook T2-weighted dynamic contrast Magnetic Resonance Lymphangiography (DCMRL) in 10 patients with Noonan syndrome and systemic anomalies. They identified central lymphatic abnormalities with retrograde intercostal flow, pulmonary lymphatic perfusion, and thoracic duct abnormalities (Biko et al., 2019).

We present the investigations and management of two patients with a severe progressive central conducting lymphatic abnormality, and, in one patient, report the therapeutic role of targeted molecular therapy with a MEK inhibitor.

## Patient A

A 26 year old man with a clinical diagnosis of RASopathy, presented with recent onset of genital and bilateral lower limb lymphoedema. Mild polyhydramnios was noted during the pregnancy. He was diagnosed with hypertrophic cardiomyopathy in infancy, which subsequently resolved. He had severe feeding difficulties necessitating feeding via a gastrostomy until the age of 4 years. He had mild to moderate learning difficulties. Epilepsy developed at puberty. There was no family history of similar problems.

**FIGURE 1:**
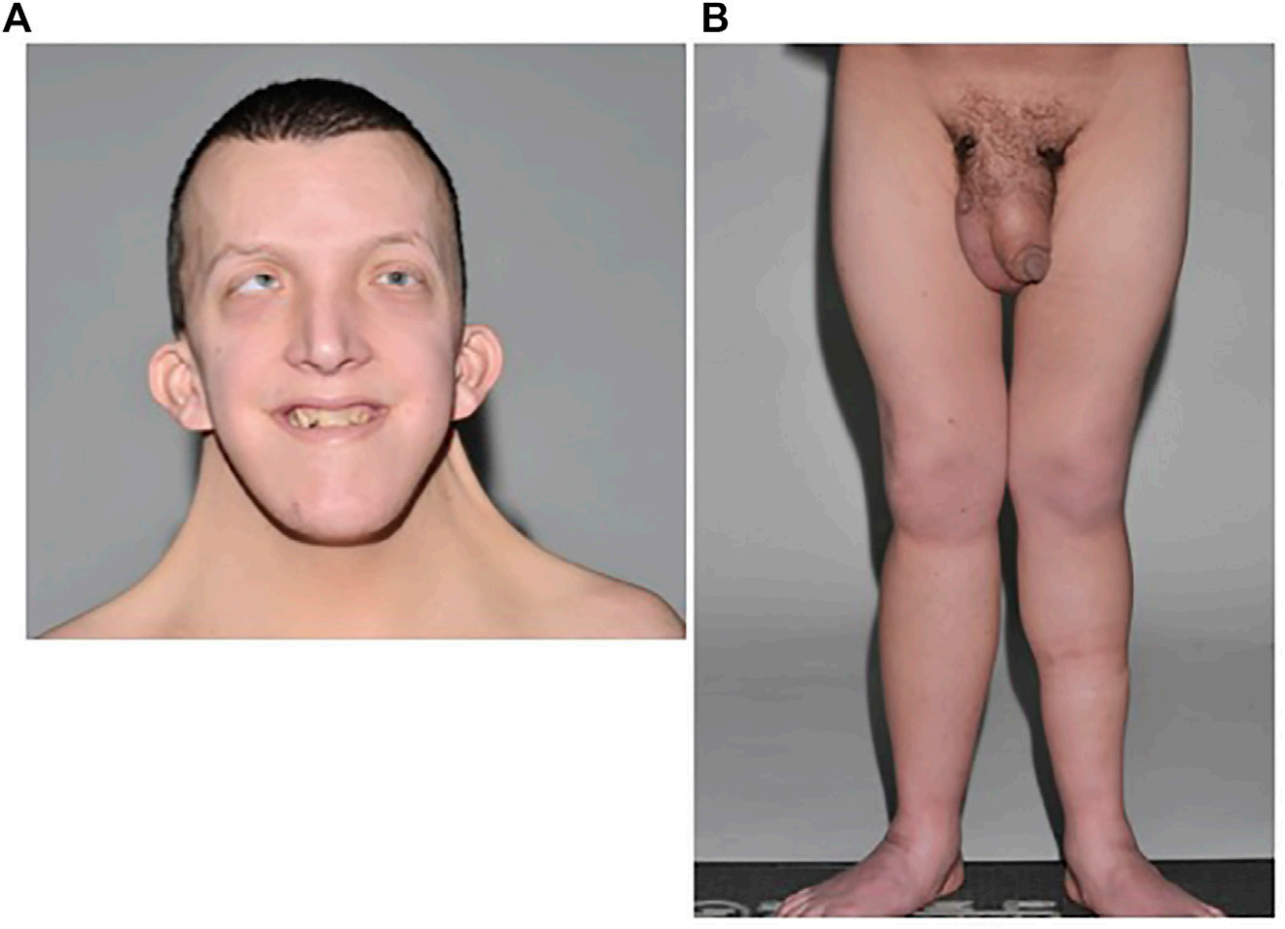
**(A)** Patient A with typical facies of Noonan syndrome, including moderate ptosis, down-slanting palpebral fissures, marked neck webbing, bilateral epicanthic folds, and low-set posteriorly rotated ears. **(B)** Bilateral lower limb lymphoedema with mild scrotal and penile oedema.

Examination revealed typical facies of Noonan syndrome (Figure 1A). He was of short stature (1.57 m, <0.4th centile), despite previous treatment with growth hormone. Pectus carinatum and excavatum were noted. Bilateral lower limb lymphoedema and mild scrotal lymphoedema was present, but no evidence of cutaneous chylous lymphangiectasia (Figure 1B).

He underwent genetic testing and a pathogenic heterozygous *de novo* variant was identified in BRAF exon 6 (c.770A>G; p.Gln257Arg).

At the age of 28 he developed bilateral pleural effusions and evidence of a protein losing enteropathy i.e., intestinal lymphangiectasia, with low albumin, low immunoglobulins and raised faecal alpha 1-antitrypsin levels. He had progressive shortness of breath due to an increasing pleural effusion of the left lung. Drainage of the left pleural effusion and a pleurodesis was performed. The fluid was confirmed to be chylous with a triglyceride level of 11.9 mmol/L in the pleural fluid (2.07 mmol/L in serum), and cholesterol level of 0.8 mmol/L (0.78 mmol/L in serum). However, subsequently he developed an increased accumulation of fluid in the right pleural cavity and increasing skin and subcutaneous oedema of his abdomen, lower limbs and genitalia. A chest drain was inserted on the right and copious amounts of milky fluid drained daily. At this point he was losing 6 L per day of chylous pleural fluid, and it proved challenging to adequately replace his electrolytes. He required a prolonged admission to the Intensive Care Unit. Initially, a low fat (MCT) diet and intravenous diuretics temporarily improved the dyspnoea, effusions and abdominal wall oedema; however, over time the observed beneficial effects abated. Three months later he underwent a right-sided pleurodesis, pleurectomy and ligation of the thoracic duct was also undertaken, but with no long-term benefit. He became increasingly malnourished and his albumin level was persistently <10 g/L. He had profound electrolyte disturbances, including hyponatraemia and hypokalaemia, as a result of extreme fluid loss into the pleural cavities.

**FIGURE 2:**
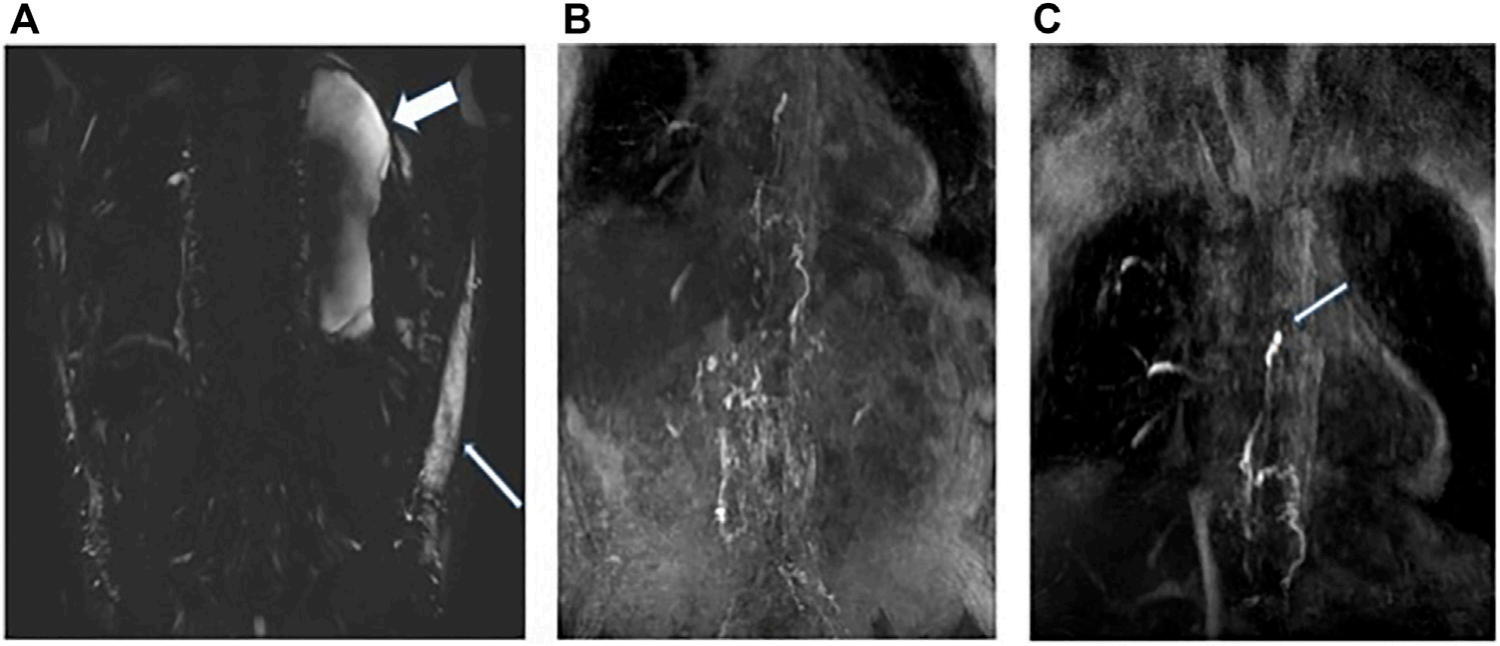
Patient A: **(A)** Heavy T2 weighted coronal MRI image demonstrating moderate left sided pleural effusion (wide white arrow) and soft tissue oedema (narrow white arrow). **(B)** Post contrast MRI coronal MIP image demonstrating bilateral flow of contrast from the inguinal lymph nodes into the retroperitoneal lymphatics and up into the distal thoracic duct. **(C)** Termination of the thoracic duct at the level of the carina (white arrow) at the site of surgical ligation. No single point of drainage into the terminal thoracic duct from the abdomen is identified. No leakage of contrast is seen into the pleural effusions.

DCMRL was performed with intranodal injection of gadolinium into the inguinal lymph nodes on both sides (Figures 2A–C). Initial T2 weighted imaging was undertaken prior to contrast and demonstrated a moderate left sided pleural effusion (Figure 2A) and marked soft tissue oedema. At the time of imaging there was a right sided chest drain *in situ*. Scanning was then re-commenced as injection of 8 ml of a Gadolinium containing contrast agent, Dotarem [(gadoterate meglumine) (Guerbet, Raleigh, United States)] was carried out on each side at a rate of 1–2 ml/min. This demonstrated bilateral drainage of contrast from the iliac systems into the cisterna chylii (Figure 2B). The thoracic duct appeared interrupted which was in keeping with the history of surgical ligation of the thoracic duct (Figure 2C: White arrow). No single lymphatic channel was seen draining the abdominal lymphatics into the neck. No leakage of contrast was seen into the pleural cavity.

Multiple management strategies were employed over a 2-year period including high-dose intravenous diuretics, octreotide, medium chain triglyceride (MCT) diet, parenteral nutrition, albumin infusions, a trial of sirolimus (which was ineffective), and high-dose systemic steroids.

None of these treatments reversed his progressive pleural fluid collection. Sadly, despite aggressive treatment, his lymphatic system continued to fail, with persistent chylothoraces and increasing oedema of his lower limbs, genitalia and abdominal wall. Two years after presenting with the pleural effusions, and after multiple prolonged hospital admissions (including one lasting for 9 months), he suffered a prolonged seizure resulting in cardiac arrest, and could not be resuscitated. A MEK inhibitor was never given as there had been no previous reports of its use for the RASopathies.

## Patient B

**FIGURE 3:**
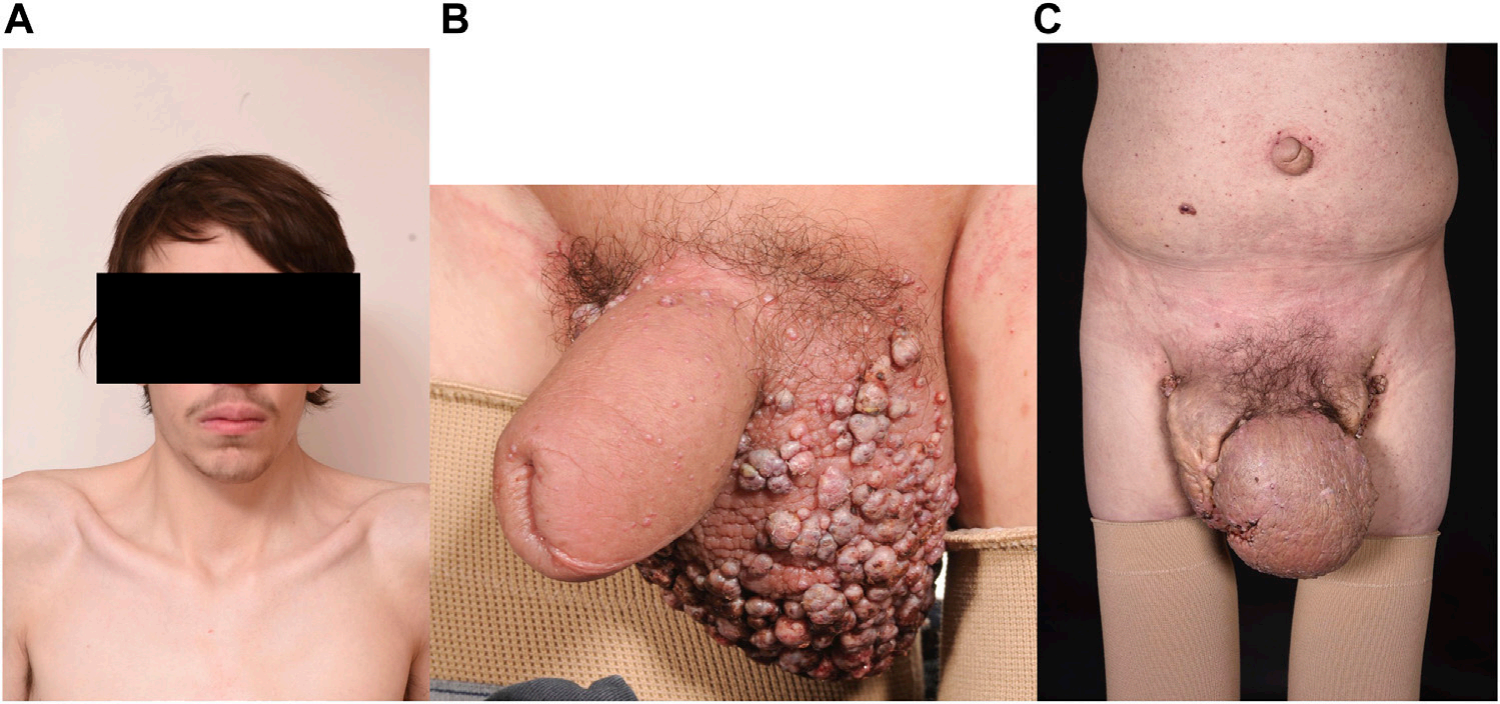
**(A)** Patient B Typical Noonan facies with hypertelorism, down slanting palpebral fissures. **(B)** Scrotal oedema age 16 years with lymphangiectactic blisters. **(C)** progressively worsening scrotal oedema age 24 years.

Patient B was known to have a diagnosis of Noonan syndrome due to a *de novo* pathogenic variant in *RIT1* [c.246T>G: p. (Phe82Leu)], with the typical facial features, a mild pulmonary stenosis at birth (not requiring surgery) but with normal intelligence and normal stature (Figure 3A). He developed lower limb lymphoedema in childhood and genital lymphoedema in his teenage years. He received compression therapy and underwent genital debulking surgery with good results.

At the age of 16 he developed chylothoraces that were successfully, but temporarily, treated with bilateral pleurodesis. There were ongoing issues with mild lower limb lymphoedema, but he was otherwise in good health, able to work and independent.

At the age of 22 he was noted to have progressive peripheral lymphoedema and his genital lymphoedema had dramatically progressed despite the use of compression garments (Figures 3B,C). Chylous reflux had complicated the genital lymphoedema with chyle leaking from the lymph blisters on his penis and scrotum on a daily basis, exacerbated by the oral intake of dietary fats. He had become short of breath at rest resulting from his increasing pericardial and pleural effusions.

**FIGURE 4:**
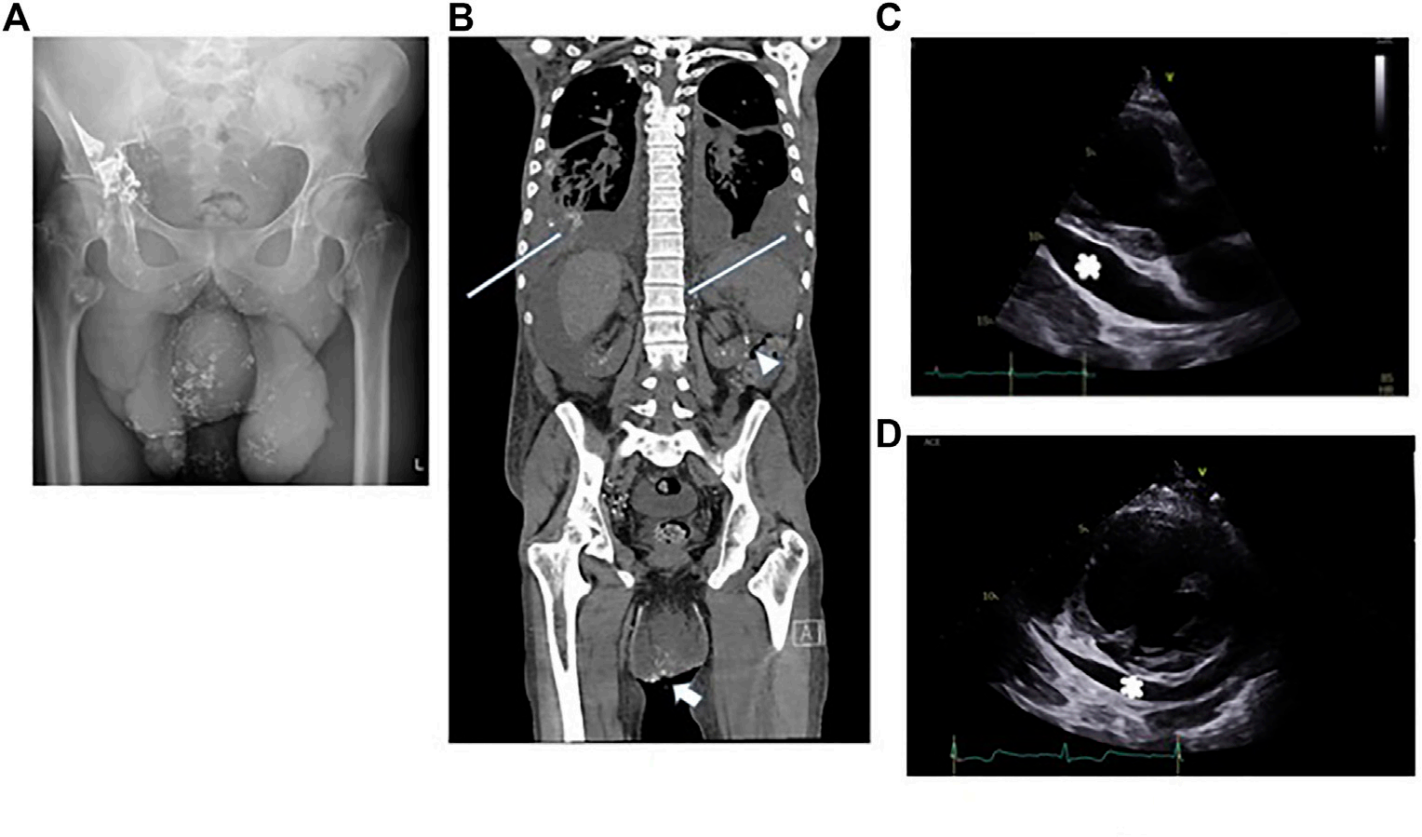
Patient B: **(A)** Reflux of contrast into the penoscrotal mass from the right inguinal lymphatics. **(B)** Non contrast CT showing bilateral pleural effusions, ascites and the penoscrotal mass. Long white arrows show residual lipiodol leakage into the pleural effusions bilaterally. Arrowhead shows lipiodol in the small bowel mesentery. Short white arrow shows lipiodol reflux into the scrotum. **(C)** Echocardiogram, 2 months after pericardiocentesis -parasternal long axis view showing a large >2 cm pericardial effusion (white asterix) adjacent to the left ventricular posterior wall. **(D)** Echocardiogram, 4 months after commencing treatment with trametinib parasternal short axis view confirming reduction in pericardial effusion size around the left ventricle (marked with a white asterix).

Despite trying to manage these effusions in the outpatient setting he required urgent admission to his local hospital for peritonitis. Treatment with high dose diuretics failed to control his symptoms. Large volumes of fluid were drained from his abdomen (3.5 L) and pericardium (1.8 L). Analysis of the fluids confirmed they were chylous: fluid albumin level 32 g/L (serum albumin 31 g/L) triglyceride level 5.9 mmol/L (serum triglyceride 1.06 mmol/L), cholesterol 2.1 mmol/L (serum cholesterol 2.17 mmol/L), protein 4.7 g/L and chylomicrons were present in the fluid. He underwent multiple investigations, including Intranodal Lymphangiography using Lipiodol. Only a single lymph node was identified that could be targeted in the right groin. The procedure was carried out under fluoroscopic guidance following initial ultrasound guided puncture of an inguinal lymph node, followed by injection of Lipiodol at a rate of approximately 0.5 ml/min. Once contrast was seen in the chest, the study was terminated, and a delayed x-ray was performed 2 h later. The initial study and the delayed x-ray confirmed reflux of lymphatic fluid into the penoscrotal mass (Figure 4A). A delayed non contrast CT scan performed 4 h later demonstrated leakage of contrast (lymphatic fluid) bilaterally into the pleural effusions and leakage or reflux of contrast into the small bowel mesentery (Figure 4B) but this was not felt to be amenable to surgery or embolisation.

Clinical examination and biochemical markers demonstrated that he was hypoxic, dyspnoeic, and malnourished as well as anaemic, hyponatraemic and hypoalbuminaemic which we consider to be indicators that conventional therapies (nutritional supplements) and rescue therapies (diuretics/mechanical drainage) were insufficient.

The transthoracic echocardiogram 2 months after pericardiocentesis demonstrated a marked increase in the size of pericardial effusion since drainage: seen largest at 2.7 cm around the basal lateral left ventricular wall and 2.9 cm around the right atrium. All echocardiographic signs of hemodynamic compromise were noted including ∼50% trans-tricuspid respiratory Doppler variation, systolic collapse of the right atrium, diastolic collapse of the basal right ventricular free wall and a dilated and fixed inferior vena cava (Figure 4C).

Furthermore, we were concerned that he was at significant risk of overwhelming sepsis, due to widespread lymphatic failure.

All standard lymphoedema treatments [compression therapy, manual lymphatic drainage massage (MLD) and MCT diet] had been maximised. Patient B’s diuretic regime was optimised (oral Bumetanide and Spironolactone). Parenteral Nutrition (PN) but was poorly tolerated by the patient and failed to prevent re-accumulation of effusions. An oral MCT diet was subsequently implemented but there was no significant reduction in systemic or peripheral fluid accumulation. Octreotide was commenced, as there were anecdotal reports of efficacy (Kuroiwa et al., 2001). This also proved ineffective.

Patient B deteriorated despite the introduction of all available drug treatments and physical drainage strategies. He had widespread lymphatic failure manifesting with ascites, pleural effusions, pericardial effusion and subsequent cardiac tamponade. Progressive lymphatic dysfunction is only a recently recognised complication of Noonan syndrome. There had been no existing clinical treatment policy for this condition, other than attempts to manage it with repeat drainage and high dose diuretics in combination with avoidance of ingestion of fats. An MDT of specialists agreed that he had a progressive deterioration and life expectancy was poor and that novel treatment therapies must be explored.

Trametinib, a MEK inhibitor used in the treatment for cancers with activation of the RAS/MAPK pathway, was commenced at a dose of 1 mg orally daily for a month, and then increased to 2 mg orally daily thereafter. Treatment response was measured by serial echocardiograms, chest radiographs and abdominal ultrasounds in combination with regular clinical examinations by specialised physicians.

The albumin level improved (29–39 g/L) reflecting an improved nutritional state. There was a total resolution of ascites within 3 months. The pericardial effusion took longer to resolve but 18 months into treatment, the fluid was considerably reduced with only small to moderate fluid (Figure 4D). Furthermore, there were no obvious echocardiographic signs of hemodynamic compromise noted.

Minor side effects were experienced from Trametinib including nausea, gastritis, constipation and eczema of the lower legs. He developed an iron deficiency anaemia [Haemoglobin level: 89 (range 120–180), mean cell volume (MCV): 68.5 (range 80–97), iron: 3 (14–30)] requiring an iron infusion after 10 months on Trametinib.

**FIGURE 5:**
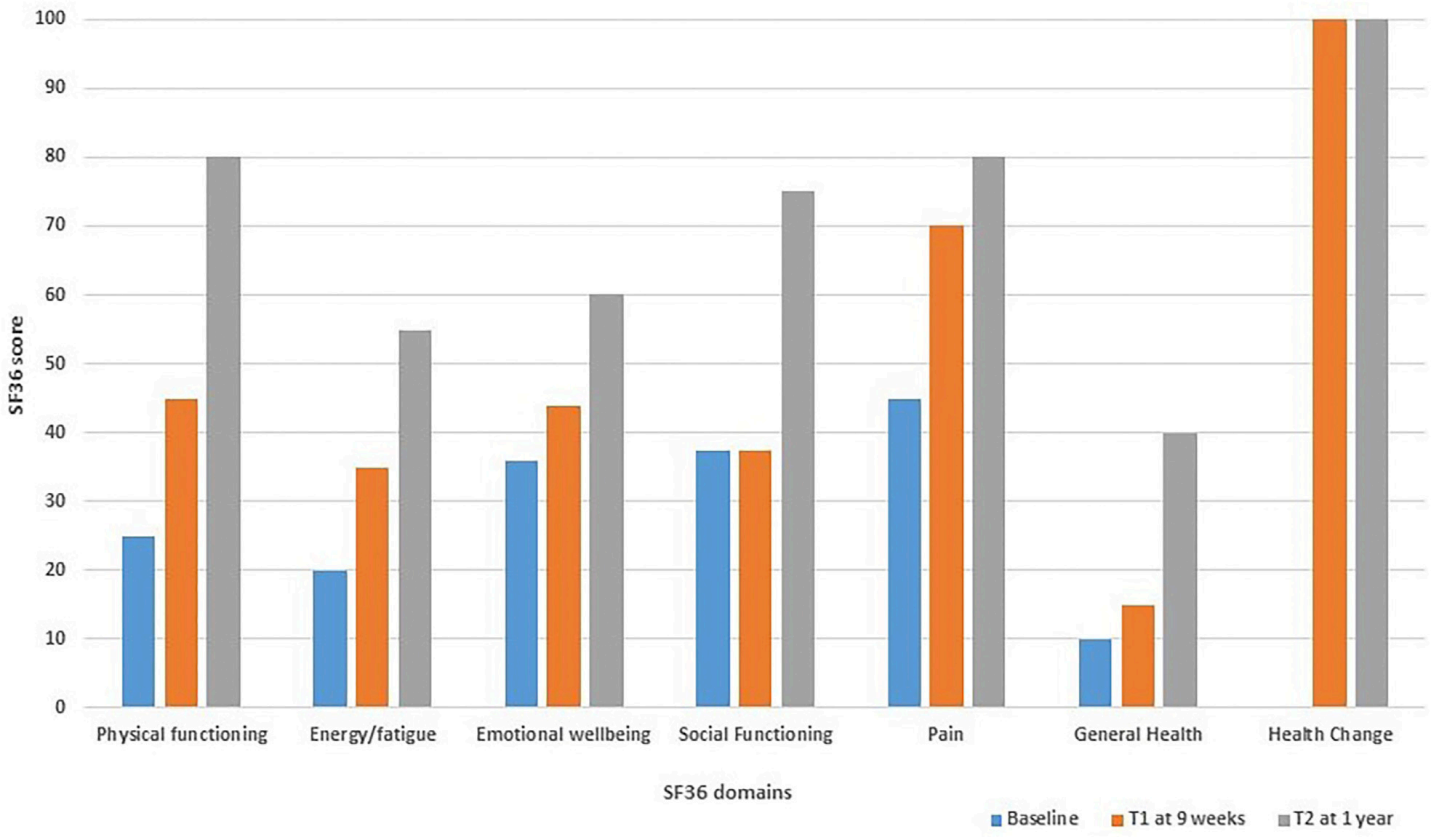
Bar chart demonstrating the 36 item Short Form (SF36) health related quality of life score for patient B; completed at baseline before Trametinib (baseline), week 9 after commencing Trametinib (T1) and at 1 year follow up (T2).

A validated self-administered online questionnaire was used as a patient outcome tool to measure the effect of the Trametinib on the patient’s general health related quality of life. The 36 item Short Form (SF36) was selected as it is commonly used for this purpose and is freely and easily available at https://orthopowertools.com/SF36 (website accessed 12/8/2021). The patient completed the SF36 at baseline before Trametinib, week 9 after Trametinib intervention and at 1 year follow up. The patient’s SF36 score improved across all domains (except limitations due to emotional health) between all time points. The most drastic improvements were in physical functioning and general health change (Figure 5).

Patient B reported a dramatic improvement in his health and returned to work. He has not required hospital admission since commencing Trametinib. The increase in activity levels, reduced subjective shortness of breath and increased oxygen saturations (93%–98% on room air) reflects an improvement in pleural and pericardial lymphatic function. These subjective and objective changes were supported by reduction of effusions.

In summary, patient B reported an improved quality of life since commencing Trametinib. Continued treatment for 28 months has avoided further hospital admissions, or drainages of effusions and resulted in an increased life expectancy. It is likely patient B will require long-term Trametinib to maintain the results of treatment so far.

## Discussion

Over the past few years, it has become increasingly observed that some patients with a RASopathy such as Noonan syndrome have progressive lymphatic failure due to a central conducting lymphatic abnormality. This initially manifests as lower limb and genital oedema, progresses to chylous reflux of the genital region and then may manifest as chylothoraces, chylopericardium, intestinal lymphangiectasia and/or ascites. The accumulation of fluid in these compartments, aggravated by the low serum albumin due to loss of fluid and protein losing enteropathy, result in clinical deterioration over a matter of months. Drainage of the fluid results in rapid re-accumulation, whilst pleurodesis or pleurectomy may lead to an accumulation of fluid elsewhere. The existing treatments (diuretics, low fat MCT diet, Octreotide) rarely improve the situation sufficiently and only provide temporary relief. Octreotide’s mechanism of action in lymphatic disease is uncertain and, in our experience, it rarely benefits patients with widespread lymphatic disease.

Understanding the molecular cause of the RASopathies has led to experimental treatment with novel targeted treatments. However, it is difficult to undertake a large-scale trial on such a rare complication. Trametinib is a highly selective reversible allosteric inhibitor of MEK1/2 activity, and is an approved treatment for cancers with activation of the RAS/MAPK pathway e.g., lung adenocarcinoma (Berger et al., 2014). Andelfinger et al., 2019 reported the use of Trametinib in two infants with Noonan syndrome associated with *RIT1* mutations, who suffered from severe, early-onset hypertrophic cardiomyopathy. One of the neonates had bilateral, chylous pleural effusions. They postulated that MEK inhibition might limit myocardial hypertrophy as *RIT1* mutations cause RAS pathway activation (Andelfinger et al., 2019). Their theory was supported by beneficial effects in preclinical mouse models for both cardiac and extra-cardiac manifestations of RASopathies (Hernandez-Porras et al., 2014; Wu et al., 2011). Andelfinger et al.’s, (2019) two patients responded well to MEK inhibition treatment as cardiac hypertrophy regressed, with sustained improvement over a total of 17 months of treatment. The chylous pleural effusions in one baby also resolved (Andelfinger, personal communication).

The use of MEK inhibitors in patients with RASopathies is further supported by the successful use of a MEK inhibitor in a patient with a similar central conducting anomaly of the lymphatic system. A gain of function somatic variant was identified in the a-rapidly accelerated fibrosarcoma (*ARAF*) gene which is also an up-regulator of the RAS-MAPKinase pathway (Li, 2019). A further child with Noonan syndrome due to a *de novo* mutation in *SOS1* and a severe lymphatic disorder was also successfully treated with trametinib (Dori et al., 2020).

The growing body of evidence that genetic conditions can be successfully treated with targeted drug therapy (McCormick et al., 2016), including successful use of MEK-inhibitors to treat complex and often inoperable neurofibromatosis type-1-associated tumours (Klesse et al., 2020), encouraged us to trial Trametinib for our patient B.

Due to the poor prognosis of patient B, whose *RIT1* mutation lies upstream of the MEK protein, there was sound reasoning to trial a MEK inhibitor. The patient responded dramatically to Trametinib with complete resolution of the ascites and a reduction in the fluid in the pleural and pericardial cavities. Prior to treatment he had had more than 3 admissions in 6 months but has not required a single admission to hospital since starting treatment with Trametinib. The initial Trametinib dose of 1 mg daily orally for a month was given to ensure tolerability, and then increased to 2 mg daily orally thereafter; which is the standard dosage when used for unresectable or metastatic melanoma and non-small cell lung cancer. Doses of Trametinib used in other reported cases of Rasopathy-related lymphatic dysfunction include 0.5–1 mg daily orally (Li et al., 2019; Dori et al., 2020) and 0.02–0.027 mg/kg/day (Andelfinger et al., 2019). The length of time patients may require MEK inhibition within this context is unknown; with reports indicating time points at publication/assessment including 6 months (Dori et al., 2020), 12 months (Li et al., 2019) and 17 months (Andelfinger et al., 2019). Our patient suffered with mild side effects, but more severe adverse effects are reported with Trametinib; including haemorrhage, retinal vein occlusion and severe skin toxicity.

Clinical understanding and decision-making have been aided by improvements in imaging of the central lymphatics, both by intranodal lymphangiography with Lipiodol and with DCMRL using gadolinium. This has enabled clear visualisation of the central lymphatics in patients with progressive CCLA and, in some patients, this may identify abnormalities that would be amenable to surgery such as lympho-venous anastomoses or interventional radiological techniques such as thoracic duct embolization and glue embolization of refluxing or leaking lymphatics. In the RASopathies, the central lymphatics become tortuous, hyperplastic and leaky. The triggers for this development are not yet understood.

In summary, we describe 2 cases with RASopathies with progressive lymphatic disease. The value of Intranodal lymphangiography for imaging, and improved understanding, of the internal lymphatic abnormalities is well demonstrated. Better understanding of the molecular cause of these rare genetic conditions has led to successful treatment with targeted drug therapy such as MEK-inhibitors (e.g., Noonan syndrome, Neurofibromatosis type 1). We demonstrate successful treatment with Trametinib in one of the cases. Targeted drug therapy will undoubtedly revolutionise how these internal lymphatic abnormalities in the RASopathies will be managed in the future.

## Data Availability

The datasets for this article are not publicly available due to concerns regarding participant/patient anonymity. Requests to access the datasets should be directed to the corresponding author.

## References

[B1] Andelfinger G. Marquis C. Raboisson M-J. Théoret Y. Waldmüller S. Wiegand G., (2019). Hypertrophic cardiomyopathy in noonan syndrome treated by MEK-inhibition. J. Am. Coll. Cardiol. 773 (17), 2237–2239. 10.1016/j.jacc.2019.01.066 PMC6916648 31047013

[B2] Berger A. H. Imielinski M. Duke F. Wala J. Kaplan N. Shi G-X., (2014). Oncogenic RIT1 mutations in lung adenocarcinoma. Oncogene 33 (35), 4418–4423. 10.1038/onc.2013.581 24469055 PMC4150988

[B3] Biko D. M. Reisen B. Otero H. J. Ravishankar C. Victoria T. Glatz A. C., (2019). Imaging of central lymphatic abnormalities in noonan syndrome. Pediatr. Radiol. 49 (5), 586–592. 10.1007/s00247-018-04337-6 30613845

[B4] Clemens R. K. Pfammatter T. Meier T. O. Alomari A. I. Amann-Vesti B. R. (2015). Combined and complex vascular malformations. Vasa. 44, 92–105. 10.1024/0301-1526/a000414 25698387

[B5] Dori Y. Smith C. Pinto E. Snyder K. March M. E. Hakonarson H., (2020). Severe lymphatic disorder resolved with MEK inhibition in a patient with noonan syndrome and SOS1 mutation. Pediatrics 146 (6), e20200167. 10.1542/peds.2020-0167 33219052

[B6] Hernandez-Porras I. Fabbiano S. Schuhmacher A. J. Aicher A. Cañamero M. Cámara J. A., (2014). K-RasV14I recapitulates Noonan syndrome in mice. Proc. Natl. Acad. Sci. U. S. A. 111 (46), 16395–16400. 10.1073/pnas.1418126111 25359213 PMC4246321

[B7] Joyce S. Gordon K. Brice G. Ostergaard P. Nagaraja R. Short J., (2015). The lymphatic phenotype in noonan and cardiofaciocutaneous syndrome. Eur. J. Hum. Genet. 23 (12), 690–696. 10.1038/ejhg.2015.175 PMC4930084 26242988

[B8] Klesse L. J. Jordan J. T. Radtke H. B. Rosser T. Schorry E. Ullrich N., (2020). The use of MEK inhibitors in neurofibromatosis type 1-associated tumors and management of toxicities. Oncologist 25 (7), e1109–e1116. 10.1634/theoncologist.2020-0069 32272491 PMC7356675

[B9] Kuroiwa G. Takayama T. Sato Y. Takahashi Y. Fujita T. Nobuoka A., (2001). Primary intestinal lymphangiectasia successfully treated with octreotide. J. Gastroenterol. 36, 129–132. 10.1007/s005350170142 11227670

[B10] Li D. March M. E. Gutierrez-Uzquiza A. Kao C. Seiler C. Pinto E., (2019). ARAF recurrent mutation causes central conducting lymphatic anomaly treatable with a MEK inhibitor. Nat. Med. 25 (7), 1116–1122. 10.1038/s41591-019-0479-2 31263281

[B11] Li D. Wenger T. Seiler C. March M. Uzquiza A. G. Kao C., (2018). Pathogenic variant in *EPHB4* results in central conducting lymphatic anomaly. Hum. Mol. Genet. 27, 3233–3245. 10.1093/hmg/ddy218 29905864 PMC7190898

[B12] McCormick A. Rosenberg S. Tier K. Balest A., (2016). A case of a central conducting lymphatic anomaly responsive to sirolimus. Pediatrics 137 (1), e20152694. 10.1542/peds.2015-2694 26729539

[B13] Opitz J. M. (1986). On congenital lymphedema. Am. J. Med. Genet. 24, 127–129. 10.1002/ajmg.1320240115 3706401

[B14] Rauen K. A. (2013). The RASopathies. Annu. Rev. Genomics Hum. Genet. 14, 355–369. 10.1146/annurev-genom-091212-153523 23875798 PMC4115674

[B15] Roberts A. E. Allanson J. E. Tartaglia M. Gelb B. D. (2013). Noonan syndrome. Lancet 381 (9863), 333–342. 10.1016/S0140-6736(12)61023-X 23312968 PMC4267483

[B16] Roberts A. E. (2001) Noonan syndrome.” in GeneReviews®. Adam M. P. Ardinger H. H. Pagon R. A., editors. Seattle, WA, U.S.A: University of Washington, Seattle; 1993. Available from: https://www.ncbi.nlm.nih.gov/books/NBK1124/

[B17] Smpokou P. Tworog-Dube E. Kucherlapati R. S. Roberts A. E. (2012). Medical complications, clinical findings and educational outcomes in adults with Noonan syndrome. Am. J. Med. Genet. A 158A, 3106–3111. 10.1002/ajmg.a.35639 23165751

[B18] Tartaglia M. Zampino G. Gelb B. D. (2010). Noonan syndrome: Clinical aspects and molecular pathogenesis. Mol. Syndromol. 1, 2–26. 10.1159/000276766 20648242 PMC2858523

[B19] Trenor C. C. Chaudry G. (2014). Complex lymphatic anomalies. Semin. Pediatr. Surg. 23, 186–190. 10.1053/j.sempedsurg.2014.07.006 25241096

[B20] Witt D. R. Hoyme H. E. Zonana J. Manchester D. K. Fryns J. P. Curry C. J. (1987). Lymphedema in noonan syndrome: Clues to pathogenesis and prenatal diagnosis and review of the literature. Am. J. Med. Genet. 27, 841–856. 10.1002/ajmg.1320270412 3321992

[B21] Wu X. Simpson J. Hong J. H. Kim K-H. Thavarajah N. K. Backx P. H. (2011). MEK-ERK pathway modulation ameliorates disease phenotypes in a mouse model of noonan syndrome associated with the Raf1(L613V) mutation. J. Clin. Invest. 121 (3), 1009–1025. 10.1172/JCI44929 21339642 PMC3049402

